# External validation of prognostic models predicting outcome after chronic subdural hematoma

**DOI:** 10.1007/s00701-022-05216-8

**Published:** 2022-05-03

**Authors:** Dana C. Holl, Ana Mikolic, Jurre Blaauw, Roger Lodewijkx, Merijn Foppen, Korné Jellema, Niels A. van der Gaag, Heleen M. den Hertog, Bram Jacobs, Joukje van der Naalt, Dagmar Verbaan, K. H. Kho, C. M. F. Dirven, Ruben Dammers, Hester F. Lingsma, David van Klaveren

**Affiliations:** 1 Department of Neurosurgery, Erasmus Medical Centre, Erasmus MC Stroke Centre, Dr Molewaterplein 40, 3015 GD Rotterdam, The Netherlands; 2 Department of Public Health, Erasmus Medical Centre, Rotterdam, The Netherlands; 3 Department of Neurology, University of Groningen, University Medical Center Groningen, Groningen, The Netherlands; 4 Department of Neurosurgery, Amsterdam Medical Centre, Amsterdam, The Netherlands; 5 Department of Neurology, Haaglanden Medical Centre, Hague, The Netherlands; 6 University Neurosurgical Centre Holland (UNCH), Leiden University Medical Centre, Haaglanden Medical Centre, Haga Teaching Hospital, Leiden, The Netherlands; 7 Department of Neurology, Isala Hospital Zwolle, Zwolle, The Netherlands; 8 Department of Neurosurgery, NeurocenterMedisch Spectrum Twente, Enschede, The Netherlands; 9 Clinical Neurophysiology Group, University of Twente, Enschede, The Netherlands

**Keywords:** Chronic subdural hematoma, Prognostic models, External validation, Recurrence, Mortality

## Abstract

**Background:**

Several prognostic models for outcomes after chronic subdural hematoma (CSDH) treatment have been published in recent years. However, these models are not sufficiently validated for use in daily clinical practice. We aimed to assess the performance of existing prediction models for outcomes in patients diagnosed with CSDH.

**Methods:**

We systematically searched relevant literature databases up to February 2021 to identify prognostic models for outcome prediction in patients diagnosed with CSDH. For the external validation of prognostic models, we used a retrospective database, containing data of 2384 patients from three Dutch regions. Prognostic models were included if they predicted either mortality, hematoma recurrence, functional outcome, or quality of life. Models were excluded when predictors were absent in our database or available for < 150 patients in our database. We assessed calibration, and discrimination (quantified by the concordance index C) of the included prognostic models in our retrospective database.

**Results:**

We identified 1680 original publications of which 1656 were excluded based on title or abstract, mostly because they did not concern CSDH or did not define a prognostic model. Out of 18 identified models, three could be externally validated in our retrospective database: a model for 30-day mortality in 1656 patients, a model for 2 months, and another for 3-month hematoma recurrence both in 1733 patients. The models overestimated the proportion of patients with these outcomes by 11% (15% predicted vs. 4% observed), 1% (10% vs. 9%), and 2% (11% vs. 9%), respectively. Their discriminative ability was poor to modest (C of 0.70 [0.63–0.77]; 0.46 [0.35–0.56]; 0.59 [0.51–0.66], respectively).

**Conclusions:**

None of the examined models showed good predictive performance for outcomes after CSDH treatment in our dataset. This study confirms the difficulty in predicting outcomes after CSDH and emphasizes the heterogeneity of CSDH patients. The importance of developing high-quality models by using unified predictors and relevant outcome measures and appropriate modeling strategies is warranted.

**Supplementary Information:**

The online version contains supplementary material available at 10.1007/s00701-022-05216-8.

## Introduction

Chronic subdural hematoma (CSDH) is a common condition in neurosurgical practice. CSDH is mainly diagnosed in older adults with an overall reported incidence ranging from 20.6 to 79.6 per 100,000 persons per year [2, 6, 24, 33]. Burr-hole craniostomy is the most commonly performed and worldwide most accepted treatment option in symptomatic CSDH [26, 41], most often with the insertion of closed-system drainage [4, 7, 8, 17, 36]. In CSDH, the multiplicity of (peri)operative options may influence the outcome after surgical treatment, in addition to the variety of outcome measures such as recurrence, mortality, functional outcome, and quality of life. However, the outcome of CSDH is not only influenced by treatment choices. The outcome can also be related to baseline characteristics such as age, sex, comorbidity, severity of symptoms, the use of medication, and the severity of abnormalities seen on baseline imaging. The contribution of various (peri)operative features to outcome is still under investigation in multiple randomized controlled trials [15].

Multivariable prognostic models are developed to predict the outcome based on baseline patient characteristics. Model-based outcome predictions can inform clinicians and patients and improve decision-making [29]. For instance, models can be used to predict the probability that a hematoma will require reoperation and hence inform the patients and their next-of-kin on what outcome to expect and which treatment option may be optimal [45]. Even if the same treatment strategy is implemented for all patients, a prognostic model can improve their management. For example, a patient with a higher probability of poor outcome can be invited for an earlier appointment or additional rehabilitation. Apart from clinical practice, prognostic models can be used for covariate adjustment in clinical trials and for standardized outcome comparisons between studies, countries, or centers [29, 52].

However, prognostic models are developed in a specific patient population and do not have to be equally successful in making predictions in another setting. Before considering the implementation of a model in clinical practice, the model should show good performance in an independent population in a different place or time [13].

Over the years, several CSDH prognostic models [1, 3, 5, 10–12, 16, 20, 22, 23, 25, 27, 30, 31, 34, 37, 38, 40, 42, 46, 48, 54–58] have been published. The developers of CSDH prognostic models aim to predict and stratify patients’ risk of mortality, recurrence, and/or functional outcome after surgical CSDH treatment. These models are developed in a specific patient population and have not been externally validated. External validation—assessing the performance of a model in a sufficiently large cohort of patients in a different place or time—is essential before these prognostic models can be considered for guiding clinical decisions. Moreover, external validation and updating of existing models are preferred before starting developing new models.

This study aims to identify existing prognostic models for outcomes after CSDH treatment and to assess the performance in a large dataset of CSDH patients.

## Material and methods

### Literature search

Medline Ovid, Embase, Web of Science, Cochrane Central, and Google Scholar were systematically searched from their starting dates to February 2021 (See Supplemental Table 1 for search string). Titles and abstracts of these studies were screened by the first author (DCH) to identify all CSDH prognostic models after which the full text was screened. Any discrepancies were discussed (authors DCH, AM, RD, and HL) and resolved through consensus.

### Selection of studies: inclusion and exclusion criteria

Studies were included if they contained at least one predictor of one of the outcomes of interest in patients with CSDH, that is mortality, recurrence, and functional status. Studies only describing possible predictors of outcome, without the development and presentation of a prediction model, were excluded. In addition, when predictors were absent in our data or were available for only a small number of patients (pre-specified minimum: 150 patients), these models were also excluded. We did not set specific quality criteria that the development studies needed to satisfy to be included.

### Data extraction

From each paper, we extracted the number of patients, inclusion criteria, predictors, outcomes, the prediction model, and its discriminative ability in the development study (area under the curve (AUC).

### Study population of the validation cohort

Independently from each other, three regions of the Netherlands (Amsterdam (AM), Rotterdam (RO), and North-East (NE)) collected retrospective data from 2384 consecutive patients who were treated for a CSDH in different time frames between 1991 and 2019. Amsterdam included 288 patients diagnosed between 2012 and 2018. In Rotterdam, two cohorts of patients were included: 509 patients diagnosed between 1991 and 2008 and 280 patients diagnosed between 2010 and 2015. North-East Netherlands included 1307 patients in this database, diagnosed between 2004 and 2019. Data were completely anonymized; all potentially identifying information was removed by the treating hospital and merged into a large retrospective database, which became the validation cohort for this external validation study.

### Measurement of predictors and outcomes in the validation cohort

Patient characteristics were extracted from clinical records.

The CSDH preoperative volume was measured with different methods. Researchers in Amsterdam used Brainlab AG (Munich, Germany) and researchers in North-East Netherlands used the ABC/2 volume formula. This formula can be used fast and easily with good accuracy [53].

One of the prognostic models used the occurrence of septations within the CSDH. The presence or absence of septations was not always available in our database. Only if a patient was diagnosed with a “trabecular” or “membranous” CSDH, information on septations was present. In other hematoma types, we could not deduce the presence of septations from the name of the hematoma type only and therefore hematoma types other than “trabecular” or “membranous” were scored as not containing septations.

Mortality within 30 days (yes/no) was determined based on the time of death.

Hematoma recurrence was defined as receiving medical treatment (reoperation or retreatment with dexamethasone) for CSDH.

### Statistical analyses

The performance of prediction models was evaluated in terms of calibration and discrimination. Calibration refers to the agreement between predicted and observed risk, and it was visualized by a calibration plot and quantified by calibration in the large (agreement between average observed and predicted outcomes and calibration intercept) and a calibration slope [50]. The calibration intercept expresses the difference between the average predicted risk and the average observed risk. An intercept > 0 indicates that predictions were on average too low, and an intercept < 0 indicates that predictions were on average too high. The calibration slope indicates if the average strength of the association between predictors and outcomes was correctly estimated. A slope < 1 indicates overfitting (overestimated associations), whereas a slope > 1 indicates underfitting (underestimated associations).

Discrimination describes the ability of a model to correctly separate patients with the outcome and without, and it was quantified by the concordance (C) index. The C-index estimates the probability that the risk prediction of randomly selected patients with the outcome (e.g., with CSDH recurrence at three months) was higher than the risk prediction of a randomly selected patient without that outcome (e.g., without CSDH recurrence at 3 months).

To understand the influence of the slope and case-mix heterogeneity on the discriminative ability of a model, we calculated the model-based concordance (mbc) [51]. The mbc is only influenced by the case-mix heterogeneity and not by the validity of regression coefficients.

Models were validated in (a) patients who had relevant data available (complete case analysis) and (b) in patients with missing predictor values imputed (imputation analysis). In imputation analysis, if a predictor variable was not assessed in a certain region, values for all patients on that variable were imputed based on available data from other hospitals. The model for multiple imputations included predictor and outcome variables, hospital regions, and auxiliary variables (e.g., hematoma thickness, aphasia, midline shift). The results were averaged over 10 imputed datasets using Rubin’s rules [35]. Missing outcomes were not imputed.

If a model was developed for a specific population (e.g., older adults), the model was validated in all patients with CSDH and in that specific subgroup (e.g., older adults). The performance of models was assessed and presented for the pooled data of all hospital centers and three separate regions in the Netherlands.

Analyses were performed in R (version 3.6.0) [32] using packages rms [18] and mice [49].

## Results

### Included publications

**Fig. 1:**
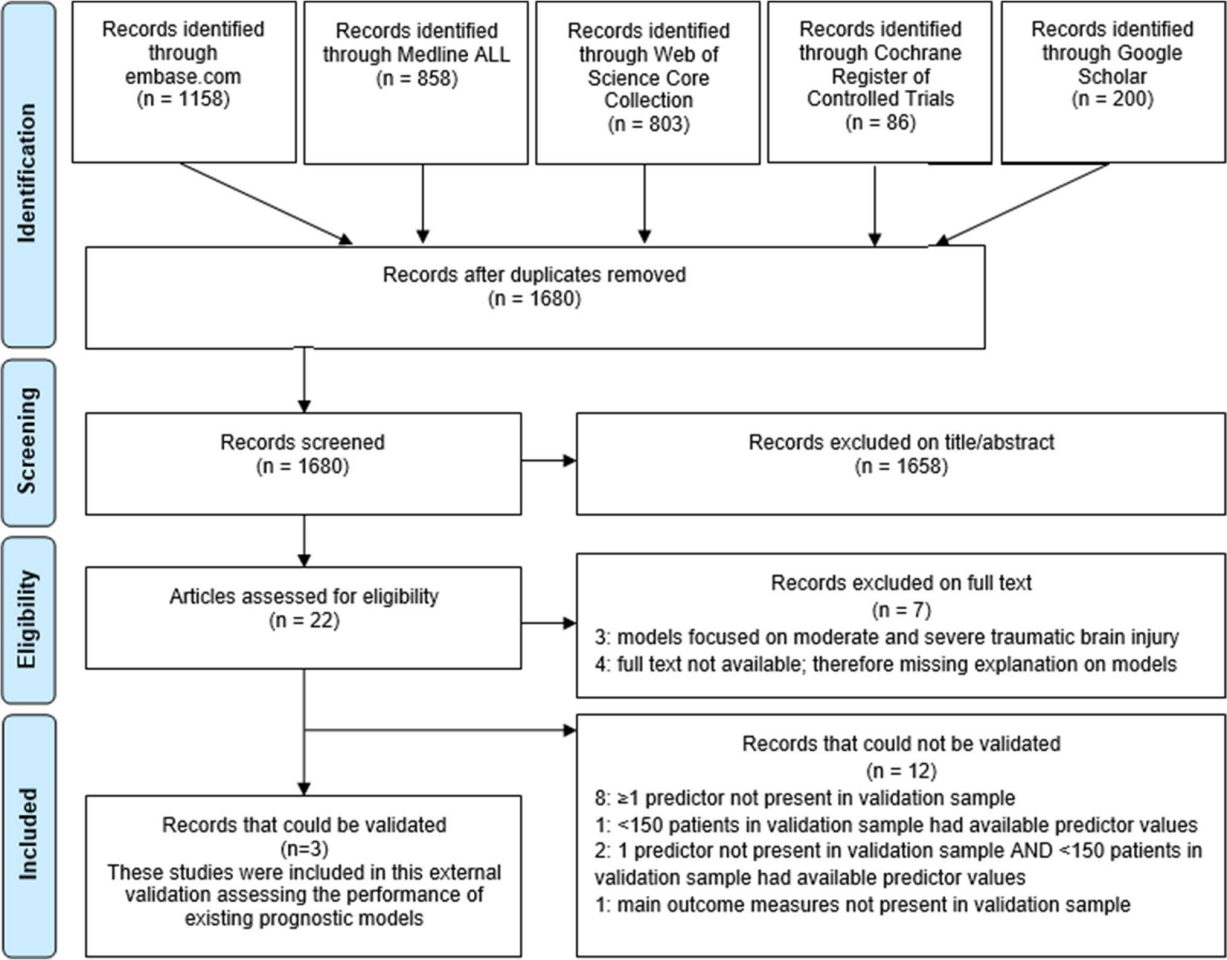
Flow diagram of the article selection process

The initial search identified 3105 studies of which 1680 remained after the removal of double references (Fig. 1).

One thousand six hundred fifty-eight records were excluded based on title/abstract because they did not concern CSDH and/or they only reported predictors of outcome, but did not develop a prognostic model. The remaining 22 articles were screened on the full text of which 7 were excluded on full text; three articles were excluded because they did focus on moderate and severe traumatic brain injury and not on CSDH specifically. Four articles were excluded because the full text was not available, and therefore, no further explanation of the prognostic models could be found.

The remaining 15 articles were included but of these 12 could not be validated (Fig. 1, Table 1).

**Table 1:** Papers presenting models that could not be validated in our data

Author, year	Model	*N*	Predictors	Outcome
Abouzari, 2009	**-**	300	Age, Glasgow Coma Scale, hematoma density, hematoma thickness, midline shift, sex, brain atrophy, intracranial air	Recurrence
Chen, 2010	**P-POSSUM**	531	Age, Glasgow Coma Scale, respiratory history, systolic blood pressure, cardiac signs, electrocardiogram, laboratory results: hemoglobin, white cell count, urea, sodium, potassium, pulse	Mortality
Chihi, 2021	**FLOP-score**	119	Age, Glasgow Coma Scale, motor deficit, brain natriuretic peptide	Functional status (modified Rankin Scale; mRS)
Maldaner, 2019	**FIT-Score**	253	Age, motor deficit, orientation	Functional status (mRS) at 3 months
McIntyre, 2020	**iGCS versus CCI**	109	Charlson Comorbidity Index (CCI), Glasgow Coma Score, 5- and 11-factor modified Frailty Index (mFI-5 and mFI-11)	Discharge location and mortality
Riemann, 2020	**-**	755	Age, comorbidities, Glasgow Coma Score, hemoglobin	Unfavorable outcome
Sastry, 2020	**-**	1647	5-factor modified Frailty Index	Complications, discharge location, readmission, and mortality
Shen, 2019	**-**	102	Use of antithrombotics, brain atrophy, pneumocephalus volume	Recurrence of bilateral CSDH
Stanisic, 2017	**Oslo CSDH Scale**	107	Density on CT-scan, preoperative CSDH volume, postoperative CSDH volume	Recurrence requiring reoperation
Won, 2019	**modified Oslo Grading System (mOGS)**	389	Density on CT-scan, preoperative CSDH volume, postoperative seizure, postoperative air trapping, postoperative CSDH volume	Postoperative recurrence
Yan, 2018	**-**	514	Age, preoperative CSDH volume, CSDH classification, postoperative CSDH volume	Postoperative recurrence
Kwon, 2018	**CSDH Scoring System**	154	Age, Glasgow Coma Scale, hematoma thickness, midline shift, motor function, orientation	Functional status (mRS) at 6 months

^*^GCS, Glasgow Coma Scale; **SDH = subdural hematoma; ^҂^*TBI*, traumatic brain injury

For eight articles, one or more predictors of the described models could not be found in our retrospective database (e.g., frailty scores, laboratory results, and pneumocephalus volume). For one article, less than 150 patients in the validation cohort had available predictor values (e.g., postoperative volume), and for two articles, one predictor could not be found in our retrospective database and another predictor had too many missing values. For one article, the main outcome measure was missing in our retrospective database. Finally, three papers (4 models) were included in the external validation (Table 2).

**Table 2:** Papers included in the external validation

Author, year	Model	Population	Operative technique	Predictors	Outcome	AUC in the development study
Alford et al., 2020	Subdural Hematoma in the Elderly (SHE) score	Age > 65 with an CSDH (*N* = 89) Retrospective data	Unknown	Age (< 80, ≥ 80), admission GCS score* (3–4, 5–12, 13–15), SDH** volume in mL (< 50, ≥ 50)	30-day mortality	0.80
Andersen et al., 2018	Model A-postoperative Model B-preoperative (in nomograms)	Unilateral CSDH, diagnosed 2010–2012 (*N* = 763). Retrospective data	Burr-hole craniostomy or craniotomy	SDH**volume, type, drainage time, subgaleal drain, surgical complications, history of hypertension	3-month recurrence requiring reoperation	Optimism-corrected C-index of 0.63 for model A; 0.60 for model B
Jack et al., 2014	Risk factor scoring system for CSDH	CSDH, diagnosed 2005–2009 (*N* = 331). Retrospective data	Burr-hole craniostomy or craniotomy	Age (≤ 80, > 80), SDH**volume in mL (≤ 160, > 160), septations in SDH**	2-month recurrence requiring reoperation	Not reported

^*^GCS, Glasgow Coma Scale; **SDH, subdural hematoma; ^҂^*TBI*, traumatic brain injury

### Models selected for validation

All selected models were developed for patients with unilateral hematoma. The Subdural Hematoma in the Elderly (SHE)-scoring model by Alford [3] was developed to predict 30-day mortality in older patients (> 65 years) based on age, admission Glasgow Coma Scale (GCS) score, and hematoma volume. The model by Jack [23] was developed to predict 2-month hematoma recurrence based on age, hematoma volume, and septations on CT. The preoperative prognostic model (model B) proposed by Andersen [5] aimed to predict 3-month recurrence based on hematoma volume, hematoma density, and history of hypertension. Andersen’s postoperative model (model A) additionally included drainage time, drain type, and surgical complications (Table 1). The Andersen models, developed with Fine-Gray regression, were validated based on predictions derived from their nomograms (Supplemental Table 2).

### Population

One thousand seven hundred sixty patients with a unilateral hematoma were included (55% from NE, 35% from RO, 11% from AM; Table 2). Four primary treatment modalities were used; 47% received surgery, 43% surgery with additional dexamethasone, 3% dexamethasone, and 7% a wait and see policy. The mean (SD) age was 73.0 years (12.4), 1293 males (74%), and the mean hematoma volume was 112 mL (cc) (54.5). Four percent of patients died within 30 days, 9% had a recurrence of CSDH requiring retreatment at 2 months, and 10% at 3 months (Table 3).

**Table 3:** Baseline characteristics, predictors, and outcomes of patients with chronic subdural hematoma

	Overall	Missing	Available 30-day mortality (Alford)	Missing	Available 2-month recurrence (Jack)	Missing	Available 3-month recurrence (Andersen)	Missing
Sample size	1760		1656		1733		1733	
Sex = male (%)	1293 (73.5)	0	1232 (74.4)	0	1276 (73.6)	0	1276 (73.6)	0
Hospital (%)		0		0		0		0
Amsterdam	186 (10.6)		87 (5.3)		159 (9.2)		159 (9.2)	
North-East	963 (54.7)		963 (58.2)		963 (55.6)		963 (55.6)	
Rotterdam	611 (34.7)		606 (36.6)		611 (35.3)		611 (35.3)	
Treatment (%)		0		0		0		0
Wait and see	129 (7.3)		129 (7.8)		129 (7.4)		129 (7.4)	
Surgery	830 (47.2)		731 (44.1)		803 (46.3)		803 (46.3)	
Surgery + dexamethasone	754 (42.8)		749 (45.2)		754 (43.5)		754 (43.5)	
Dexamethasone	47 (2.7)		47 (2.8)		47 (2.7)		47 (2.7)	
Predictors								
Age (mean (SD))	73.0 (12.4)	0	73.0 (12.4)	0	72.9 (12.4)	0	72.9 (12.4)	0
Hypertension (%)	218 (16.1)	22.9	X		X		X	
Baseline Glasgow Coma Scale score (%)		6.5		6.4	X		X	
< 5	4 (0.2)		4 (0.3)					
5–12	195 (11.8)		192 (12.4)					
13–15	1447 (87.9)		1354 (87.4)					
Baseline total volume (mL) (mean (SD))	112.6 (54.5)	46.6	111.3 (54.8)	47.8	112.8 (54.7)	46.9	112.8 (54.7)	46.9
Baseline septations (%)	335 (37.2)	48.8	X		328 (37.1)	49	X	
Density (%)		45.4	X		X			46
Homogeneous	503 (52.3)						487 (52.0)	
Membranous	331 (34.4)						325 (34.7)	
Mixed	4 (0.4)						3 (0.3)	
Separated	123 (12.8)						121 (12.9)	
Drainage time (%)		56.9	X		X			57.2
No drain	100 (13.2)						98 (13.2)	
1–24 h	385 (50.7)						373 (50.3)	
24–48 h	205 (27.0)						202 (27.2)	
> 48 h	69 (9.1)						69 (9.3)	
Surgery drain type (%)		74.9	X		X			75.7
No drain	100 (22.6)						98 (23.3)	
Subdural	163 (36.9)						147 (34.9)	
Subgaleal/subperiosteal	179 (40.5)						176 (41.8)	
Postoperative surgical complications (%)	99 (7.2)	22.4	X		X		95 (7.1)	22.7
Outcomes								
Mortality 30 days (%)	65 (3.9)	5.9	65 (3.9)	0	X		X	
Recurrence within 2 months (%)	155 (8.9)	1.5	X		155 (8.9)	0	X	
Recurrence within 3 months = 1 (%)	164 (9.5)	1.5	X			X	164 (9.5)	0

X = not in the model; *SD*, standard deviation; mL, milliliters

### Performance of models in the retrospective database

One thousand six hundred fifty-six patients with available information on 30-day mortality were selected for validation of Alford’s model and 1733 patients with available information on 2-month and 3-month hematoma recurrence were selected for validation of Jack’s model and Andersen’s models, respectively.

**Fig. 2:**
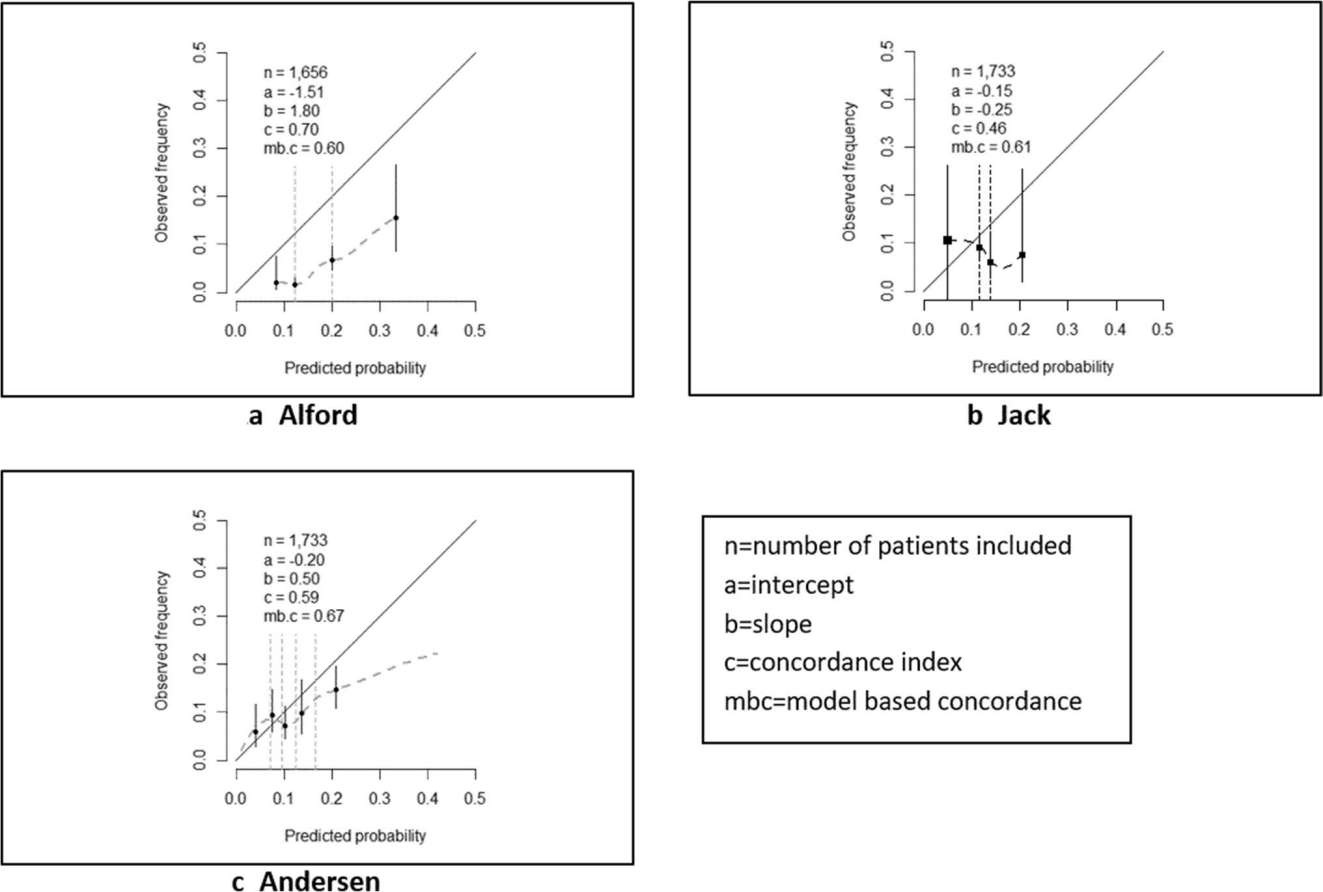
Performance of models in the retrospective database. **a** Alford. **b** Jack. **c** Andersen

The prognostic model of Alford predicted that 15% of patients would die within 30 days, whereas the observed proportion in our data was 4%. Thus, it overestimated the proportion of patients dying within 30 days by 11 percentage points (intercept =  − 1.51 [− 1.77, − 1.26]; Fig. 2a, Table 4).

**Table 4:** Performance of models (Alford, Jack, and Andersen) in external validation: complete case and imputation analyses

	Alford model	Jack model	Andersen model	
			Model A	Model B
Original C	0.80	x	0.63*	0.60*
Mbc	0.60	0.61	0.72	0.67
Complete case analysis				
N/event number	823/23	852/79	22/3	782/77
M predicted; M observed (%)	15%; 2.8%	15%; 2.8%	15%; 2.8%	15%; 2.8%
Intercept	− 1.84 [− 2.25, − 1.42]	− 0.04 [− 0.27, 0.19]	− 0.63 [− 1.90, 0.65]	− 0.25 [− 0.49, − 0.01]
C	0.70 [0.59–0.82]	0.48 [0.43–0.54]	0.67 [0.31–1.02]	0.60 [0.54–0.67]
Slope	1.92 [0.99, 2.85]	− 0.02 [− 0.47, 0.43]	0.69 [− 0.85, 2.22]	0.63 [0.25, 1.02]
Imputation analysis				
N/event number	1656/65	1733/155	1733/164	1733/164
M predicted-, M observed (%)	15.4%, 3.9%	10.2%, 8.9%	13.9%, 9.5%	11.3%, 9.5%
Intercept	− 1.51 [− 1.77, − 1.26]	− 0.15 [− 0.33, 0.02]	− 0.46 [− 0.73, − 0.19]	− 0.20 [− 0.44, 0.05]
C	0.70 [0.63–0.77]	0.46 [0.35–0.56]	0.65 [0.57–0.73]	0.59 [0.51–0.66]
Slope	1.80 [1.17, 2.43]	− 0.25 [− 1.05, − 0.56]	0.64 [0.18, 1.11]	0.50 [0.01, 1.00]

^*^Corrected for optimism. C, concordance index; M, mean; Mbc, model-based concordance; *N*, sample size

The overestimation of the 30-day mortality rate was consistent for the patient selection (> 65 years) that was used for model development (16% predicted vs. 5% observed; intercept =  − 1.38 [− 1.65, − 1.12]); Supplemental Table 2). The slope (1.92 [0.99, 2.85]) indicated a stronger association between the predictors and the outcome in our data. Nevertheless, the discriminative ability (C = 0.70 [0.63–0.77]) was reduced by the more homogeneous case-mix in our study (mbc = 0.60 versus C = 0.80 in the development study).

The prognostic model by Jack (2-month hematoma recurrence) showed a negative calibration slope, indicating reverse predictor effects (− 0.25 [− 1.05, − 0.56]). This indicated that higher predicted probabilities of recurrence by the model were in our data associated with lower observed rates. Additional analyses showed, for instance, that, in contrast with the model, age above 80 was associated with a lower likelihood of recurrence at 2 months in our data (Supplemental Fig. 1).

The proportion of patients with recurrent hematoma by 3 months was estimated accurately (10% predicted vs. 9% observed; intercept =  − 0.15 [− 0.33, 0.02]), but the discriminative ability of the prognostic model was extremely poor (C < 0.50; Table 4; Fig. 2b).

Andersen’s preoperative model (3-month hematoma recurrence) (B) slightly overestimated the proportion of patients with a recurrence within 3 months (11% predicted vs. 9% observed; intercept =  − 0.20 [− 0.44, 0.05]). The calibration slope indicated that the effect of the predictors on outcomes in the validation data was much weaker than in the model (calibration slope = 0.50 [0.01, 1.00]; Table 4). The discriminative ability of model B (C = 0.59 [0.51–0.66]), corresponded to the development study (C = 0.60), but also reflected the more heterogeneous case-mix in the validation study (mbc = 0.67; Fig. 2c).

The performance of Andersen’s postoperative model (3-month hematoma recurrence) (A) was assessed with great uncertainty due to a large amount of missing data in postoperative variables (e.g., type of drain). The effects of predictors were weaker in the validation study (calibration slope = 0.64 [0.18, 1.11]) and the model overestimated the proportion of patients with 3-month recurrence (14% predicted vs. 9% observed; intercept =  − 0.46 [− 0.73, − 0.19]). It showed a slightly higher discriminative ability (0.65 [0.57–0.73]) than in the development study (C = 0.63), lifted by a more heterogeneous case-mix than in the development study.

The results of complete case analyses were consistent with imputation analyses (Table 4). In addition, analyses per hospital region generally showed consistent results (Supplemental Table 3).

## Discussion

We examined the performance of three published prognostic models for the prediction of outcomes in patients with unilateral CSDH using a retrospective database, which contains data from three regions in the Netherlands. None of the models showed both good discriminative ability and calibration in our data. The most likely explanations of the predictive performance of the models in our data concern suboptimal modeling strategies and differences in study populations.

The differences in the population (case-mix) and differences in the distribution of predictors (case-mix heterogeneity) between the development and validation study can affect model performance in the validation setting. The prognostic model by Alford [3] largely overestimated the percentage of patients who died within 30 days, which could be associated with the substantially different mortality rate between the development study and validation study. It is possible that the patient population was more severely affected in the development study, which was not captured by the predictors in the model; for instance, patients might have had more comorbidities. In addition, although this model was able to discriminate reasonably well between patients who died and did not die within 30 days based on age, hematoma volume, and GCS score, the discrimination ability was decreased by the more homogeneous case-mix in our data. The case-mix and case-mix heterogeneity of the validation data also differed compared to the study of Andersen; for instance, patients had a higher GCS score, a smaller hematoma volume, a lower percentage of drain placement, and a different distribution of hematoma density [5]. In our retrospective validation cohort dataset, almost half of the patients were treated with dexamethasone; 43% of patients were operated with additional dexamethasone and 3% received primary dexamethasone. In these patients, the recurrence rate might be lower, but also the favorable outcome is expected to be worse and patients in the validation cohort might suffer from more adverse events and higher mortality in comparison to the cohorts used for model development [21].

Moreover, the effects of predictors differed between our study and development studies. For instance, whereas older age was predictive of 2-month recurrence in the model of Jack [23], in our dataset, age above 80 was associated with a lower recurrence rate. It is possible that older patients were more likely to die or to receive no treatment at all, in case of hematoma recurrence or in case of comorbidity or greater frailty scores in the validation study. However, frailty scores were not included in the retrospective database. In addition, different definitions of predictors could have contributed to observed differences in the effects of predictors. For example, the inter-rater variability concerning the classification of hematoma types is considered low [47], but assessing septations on a CT-scan is prone to inter-rater variability because membranes cannot always be clearly recognized on CT-scans [39]. If the predictor “septations” was not specifically scored in patients, trabecular hematomas were marked as “septations present.” All other hematoma types (homogenous, mixed, and separated) were marked as “septations absent.” This restraint in detecting septations in the validation cohort is expected to lead to an underestimation of septations in our population, because septations can also occur in homogenous, mixed, and separated hematoma types.

Finally, suboptimal modeling strategies have likely negatively affected the effects of predictors and model performance in a new setting (our data). A very small sample size of older adults with CSDH was used for the development of the Alford model [31]. In addition, in the models of Alford [31] and Jack [23], continuous predictors were dichotomized/categorized (e.g., age, hematoma volume). Although categorization can make a model seem appealing and easier to use, it leads to a loss of information and usually poor performance in other cohorts [28, 43]. Furthermore, the predictors were selected based on *p*-values and there was no internal validation, which lead to overfitting; meaning that predictor effects are overestimated, model performance in the development sample is overoptimistic, and performance in external validation is poor(er) [44]. The authors of the Andersen [5] model did apply shrinkage in the model development, an approach to prevent overfitting, but the models still showed weaker effects of predictors in our study, probably due to differences in case-mix. In addition, the discriminative ability of this model was also modest in the development study (C = 0.60). The generally limited discriminative ability obtained in both the development study and validation cohort suggests that other variables could be considered for the prediction of this outcome in future studies.

Besides considering other predictors, the strategies for developing models for predicting outcome after CSDH should therefore be improved. Future studies should comprise large samples and collaborative efforts. The predictors should not be primarily selected by *p*-values but based on level I evidence and clinical expertise. Also, internal validation should be applied in model development. In that way, the effects of predictors are less likely to be exaggerated leading to optimistic model performance [44]. The categorization of continuous variables should be avoided and missing values should be imputed using single or multiple imputation techniques [28, 43]. Unified definitions of baseline data elements (predictors) and a unified core outcome set would also facilitate a more reliable establishment, validation, and clinical usefulness of models. In addition, when proposing a new prognostic model, all relevant information that indicates model performance and enables future external validation studies should be reported, such as the full model equation and discriminative ability.

Furthermore, the results also suggest that it is difficult to predict the outcome after CSDH. It is known that “there is significant heterogeneity in the data elements that are collected and reported as part of clinical studies examining outcomes for CSDH” [9]. Moreover, the disease CSDH itself is also heterogeneous. CSDH patients have in common that they are generally older and that most have a high GCS score on admission, but many other characteristics differ such as frailty and overall clinical status. From our experience, a more voluminous CSDH does not necessarily indicate a larger midline shift or more severe clinical symptoms. Also, a less voluminous hematoma does not always result in a rapid recovery without the occurrence of a recurrence. Moreover, the use of anticoagulants is not necessarily related to a more voluminous CSDH, and more severe symptoms at admission are not necessarily related to a poorer functional outcome. This heterogeneity in the data of CSDH patients makes prediction inherently challenging.

### Limitations and future directions

In this study, we systematically searched for published models for the prediction of outcomes after CSDH and validated eligible models in our multicenter database. However, we did not perform a systematic review nor assessed the quality of published studies since we considered the validation as “proof” of validity. However, since our retrospective database was originally not built to validate these prognostic models, a substantial number of models could not be validated in our data due to unmeasured predictors and outcomes and due to a large number of missing values. We nevertheless describe these models and encourage other studies with available data to validate all models identified by our search. Also, for the models we did validate there was a significant percentage of missing data. Although complete case and imputation analyses point in the same direction, this should be noted as a limitation. Moreover, although we systematically searched the literature to identify existing models, finally, we did not consider the number of outcomes and our data-quality insufficient to develop a new model.

Even if we would have used a prospective database, there are no well-established predictors and outcomes derived from level I evidence. Currently, there is no consensus on the definition of CSDH and no consensus on baseline data elements nor a core outcome set. The CODE-CSDH group established a Delphi survey to reach a consensus on a core outcome set and baseline data elements to be used in future CSDH studies [19]. Results of this survey are expected in the spring of 2022. It is expected that these results will be a first step in decreasing the heterogeneity and with that improving the quality of available CSDH data. The Dutch Subdural Hematoma Research group (DSHR) [14] is planning to establish a prospective, observational, multicenter registry. Once consensus is reached on the Delphi survey, the DSHR will incorporate the baseline data elements and core outcome set in their prospective database. In the future, this prospective registry can be used for the development of a new prognostic model. This future model should predict endpoints that are relevant for clinical practice. These endpoints will correspond to the core outcome set, as to be determined at the consensus meeting of the CODE-CSDH group.

## Conclusion

Published models for the prediction of outcomes following CSDH did not perform well in our retrospective database. The study confirms the complexity of predicting outcomes in patients with CSDH and the need for the collection of standard baseline variables and a core outcome set and for improved modeling strategies, which will improve current prognostic models. This should be part of the focus of future large-scale data collections.

## Supplementary Information

Below is the link to the electronic supplementary material.

Supplementary file1 (PNG 10 KB)

Supplementary file2 (DOCX 17 KB)

Supplementary file3 (DOCX 15 KB)

Supplementary file4 (DOCX 15 KB)

## Abbreviations

CSDH: Chronic subdural hematoma
CODE-CSDH group: Core Outcomes and common Data Elements in Chronic Subdural Hematoma
DSHR: Dutch Subdural Hematoma Research group
