# Supplementary figures and images for: External validation of prognostic models predicting outcome after chronic subdural hematoma
Source: Acta Neurochir (Wien). 2022 May 3;164(10):2719–30. doi: 10.1007/s00701-022-05216-8 (PMC9519711; doi:10.1007/s00701-022-05216-8)

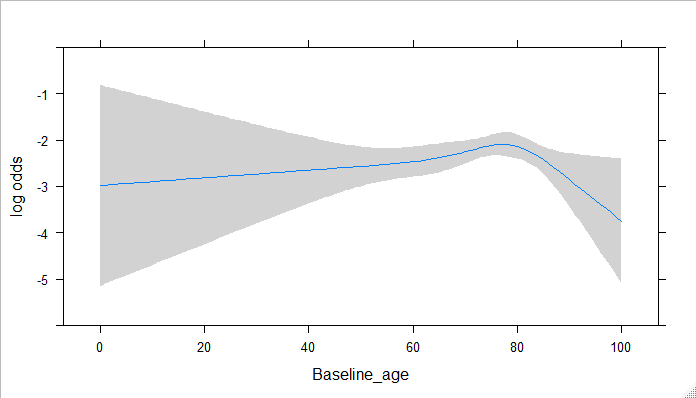

Supplement: Supplementary file 1 — Supplementary file1 (PNG 10 KB) [file 701_2022_5216_MOESM1_ESM.png]
