# Supplementary material for: External validation of prognostic models predicting outcome after chronic subdural hematoma
Source: Acta Neurochir (Wien). 2022 May 3;164(10):2719–30. doi: 10.1007/s00701-022-05216-8 (PMC9519711; doi:10.1007/s00701-022-05216-8)
Supplement: Supplementary file 2 — Supplementary file2 (DOCX 17 KB) [file 701_2022_5216_MOESM2_ESM.docx]

**Supplemental table 1**; Search terms for trials databases.

| **embase.com**  1971 - Present | ('scoring system'/de OR 'grading system'/de OR 'prognosis'/de OR ('prediction'/exp AND ('treatment outcome'/exp OR 'mortality'/exp OR 'survival'/exp OR 'recurrent disease'/de OR 'recurrence risk'/de)) OR (grading OR (scoring NEAR/3 system*) OR prognos* OR ((predict* OR associat* OR factor* OR affect* OR influen* OR impact*) NEAR/6 (outcome OR mortalit* OR recur* OR reinterven* OR re-interven* OR surviv*))):ab,ti,kw) AND ('subdural hematoma'/exp/mj OR (((subdural*) NEAR/6 (hematoma* OR haematoma* OR hemorrhag* OR haemorrhag* OR bleeding)) OR ((haemorrh* OR hemorrh*) NEAR/3 (pachymeningit* OR pachy-meningit*))):ab,ti) NOT (('acute disease'/exp OR (acute*):ab,ti,kw) NOT ('chronic disease'/de OR (chronic*):ab,ti,kw)) NOT (juvenile/exp NOT adult/exp) NOT ('case report'/exp OR 'case study'/de OR ((case NEXT/1 (report* OR stud*))):ab,ti,kw) NOT ('spinal cord hemorrhage'/exp OR (spin* NEAR/3 (hematoma* OR haematoma* OR hemorrhag* OR haemorrhag* OR bleeding)):ab,ti,kw) |
| --- | --- |
| **Medline ALL**  1946 - Present | (prognosis/ OR (Forecasting/ AND (exp treatment outcome/ OR exp mortality/ OR survival/ OR Recurrence/)) OR (grading OR (scoring ADJ3 system*) OR prognos* OR ((predict* OR associat* OR factor* OR affect* OR influen* OR impact*) ADJ6 (outcome OR mortalit* OR recur* OR reinterven* OR re-interven* OR surviv*))).ab,ti,kf.) AND (* Hematoma, Subdural/ OR Hematoma, Subdural, Chronic/ OR (((subdural*) ADJ6 (hematoma* OR haematoma* OR hemorrhag* OR haemorrhag* OR bleeding)) OR ((haemorrh* OR hemorrh*) ADJ3 (pachymeningit* OR pachy-meningit*))).ab,ti,kf.) NOT (((acute*).ab,ti,kf.) NOT ((chronic*).ab,ti,kf.)) NOT ((exp infant/ OR exp child/ OR adolescent/) NOT adult/) NOT (Case Reports/ OR ((case ADJ (report* OR stud*))).ab,ti,kf.) NOT (Hematoma, Subdural, Spinal/ OR (spin* ADJ3 (hematoma* OR haematoma* OR hemorrhag* OR haemorrhag* OR bleeding)).ab,ti,kf.) |
| **Cochrane Central Register of Controlled Trials**  1992 - Present | ((grading OR (scoring NEAR/3 system*) OR prognos* OR ((predict* OR associat* OR factor* OR affect* OR influen* OR impact*) NEAR/6 (outcome OR mortalit* OR recur* OR reinterven* OR re NEXT interven* OR surviv*))):ab,ti,kw) AND ((((subdural*) NEAR/6 (hematoma* OR haematoma* OR hemorrhag* OR haemorrhag* OR bleeding)) OR ((haemorrh* OR hemorrh*) NEAR/3 (pachymeningit* OR pachy NEXT meningit*))):ab,ti) NOT (((acute*):ab,ti,kw) NOT ((chronic*):ab,ti,kw)) NOT ((juvenile OR child* OR infan* OR adolescen*) NOT (adult*)) NOT (((case NEXT/1 (report* OR stud*))):ab,ti,kw) NOT ((spin* NEAR/3 (hematoma* OR haematoma* OR hemorrhag* OR haemorrhag* OR bleeding)):ab,ti,kw) |
| **Web of Science Core Collection**  1975 - Present | TS=(((grading OR (scoring NEAR/2 system*) OR prognos* OR ((predict* OR associat* OR factor* OR affect* OR influen* OR impact*) NEAR/5 (outcome OR mortalit* OR recur* OR reinterven* OR re-interven* OR surviv*)))) AND ((((subdural*) NEAR/5 (hematoma* OR haematoma* OR hemorrhag* OR haemorrhag* OR bleeding)) OR ((haemorrh* OR hemorrh*) NEAR/2 (pachymeningit* OR pachy-meningit*)))) NOT (((acute*)) NOT ((chronic*))) NOT ((juvenile OR child* OR infan* OR adolescen*) NOT (adult*)) NOT (((case NEAR/1 (report* OR stud*)))) NOT ((spin* NEAR/2 (hematoma* OR haematoma* OR hemorrhag* OR haemorrhag* OR bleeding)))) |
| **Google Scholar**  200 top-ranked | "Grading\|scoring system\|systems" "subdural hematoma\|haematoma\|hemorrhage\|haemorrhage\|bleeding" acute 'Grading\|scoring system\|systems' 'subdural hematoma\|haematoma\|hemorrhage\|haemorrhage\|bleeding' acute |
