# Supplementary material for: External validation of prognostic models predicting outcome after chronic subdural hematoma
Source: Acta Neurochir (Wien). 2022 May 3;164(10):2719–30. doi: 10.1007/s00701-022-05216-8 (PMC9519711; doi:10.1007/s00701-022-05216-8)
Supplement: Supplementary file 3 — Supplementary file3 (DOCX 15 KB) [file 701_2022_5216_MOESM3_ESM.docx]

**Supplemental Table 2.** The Andersen models, developed with Fine-Gray regression, were validated based on predictions derived from their nomograms

**Derived formula: Model A**

points = -74.7 + 21.6*2log(size) + 24.0*separated + 5.6*membranous + 10.4*homogenous + 9.7*(time 24-47) + 24.5*(time 1-23) + 39.4*(no drain) + 11.8*(surgical drain) + 20.1*(surgical complications) + 11.8*hypertension

probability = 1 - exp (-exp( -5.71 + 0.0346*points))

**Derived formula: Model B**

points = -74.7 + 21.6*2log(size) + 23.6*separated + 8.9*membranous + 8.9*homogenous + 10.5*hypertension

probability = 1 - exp (-exp( -4.73 + 0.0317*points))
