# Supplementary material for: External validation of prognostic models predicting outcome after chronic subdural hematoma
Source: Acta Neurochir (Wien). 2022 May 3;164(10):2719–30. doi: 10.1007/s00701-022-05216-8 (PMC9519711; doi:10.1007/s00701-022-05216-8)
Supplement: Supplementary file 4 — Supplementary file4 (DOCX 15 KB) [file 701_2022_5216_MOESM4_ESM.docx]

**Supplemental Table 3.** Performance of models (Alford, Jack and Andersen) in external validation in all patients, selected populations and different hospital regions (AM=Amsterdam, RO=Rotterdam, NE NL= North-east of the Netherlands): complete case and imputation analyses

|  | **Complete case analysis** | | | | **Imputation analysis** | | | | |
| --- | --- | --- | --- | --- | --- | --- | --- | --- | --- |
|  | **N/ event number** | **C** | **Slope** | **Intercept** | **N/ event number** | **Mbc** | **C** | **Slope** | **Intercept** |
| **Alford model, outcome: 30 day mortality, C in development=0.80** | | | | | | | | | |
| All | 823/ 23 | 0.70 [0.59-0.82] | 1.92[0.99,2.85] | -1.84[-2.25,-1.42] | 1656/65 | 0.60 | 0.70[0.63-0.77] | 1.80[1.17,2.43] | -1.51[-1.77,-1.26] |
| >65 | 656/22 | 0.70[0.59-0.81] | 1.86[0.88,2.84] | -1.71[-2.14,-1.28] | 1294/61 | 0.6 | 0.68[0.61,0.75] | 1.61[0.96,2.26] | -1.38[-1.65,-1.12] |
| AM | 59/2 | 0.81[0.73-0.88] | 1.98[-1.07,5.03] | -1.68[-3.09,-0.26) | 87/4 | 0.60 | 0.84[0.76-0.93] | 2.73[0.27-5.19] | -1.35[-2.36,-0.34] |
| RO | 0 | n/a | n/a | n/a | 606/34 | 0.61 | 0.66[0.56-0.76] | 1.32[0.48-2.16] | -1.17[-1.54,-0.81] |
| NE NL | 764/21 | 0.69[0.57-0.82] | 1.91[0.93,2.89] | -1.85[-2.29,-1.42] | 963/27 | 0.60 | 0.72[0.62-0.83] | 2.18[1.28-3.09] | -1.84[-2.22,-1.45] |
| **Jack model, outcome: 2 month recurrence** | | | | | | | | | |
| All | 852/79 | 0.48[0.43-0.54] | -0.02[-0.47,0.43] | -0.04[-0.27,0.19] | 1733/155 | 0.61 | 0.46[0.35-0.56] | -0.25[-1.05,-0.56] | -0.15[-0.33,0.02] |
| AM | 70/4 | 0.49[0.22-0.76] | -0.14[-1.98,1.70] | -0.52[-1.54,0.50] | 159/8 | 0.64 | 0.51[0.29-0.64] | -0.02[-1.53,1.58] | -0.65[-1.37,0.07] |
| RO | 0 | n/a | n/a | n/a | 611/56 | 0.58 | 0.42[0.18-0.67] | -0.58[-2.9-1.74] | -0.23[-0.54,0.09] |
| NE NL | 782/75 | 0.48[0.42-0.54] | -0.02[-0.48,0.44] | -0.01[-0.25,0.23] | 963/91 | 0.62 | 0.47[0.40,0.53] | -0.14[-0.66,0.38] | -0.04[-0.25,0.18] |
| **Andersen Model A Postoperative, outcome: 3 month recurrence, C in development 0.63*** | | | | | | | | | |
| All | 22/3 | 0.67[0.31-1.02] | 0.69[-0.85, 2.22] | -0.63[-1.90,0.65] | 1733/164 | 0.72 | 0.65[0.57-0.73] | 0.64[0.18,1.11] | -0.46[-0.73,-0.19] |
| AM | 0 | n/a | n/a | n/a | 159/11 | 0.68 | 0.82[0.67-0.96] | 2.07[0.80,3.34] | -1.29[-1.94,-0.63] |
| RO | 0 | n/a | n/a | n/a | 611/59 | 0.72 | 0.74[0.57-0.90] | 1.05[-0.01,2.11] | -0.12[-0.87,0.62] |
| NE NL | 22/3 | 0.67[0.31-1.02] | 0.69[-0.85, 2.22] | -0.63[-1.90,0.65] | 963/94 | 0.71 | 0.59[0.51-0.67] | 0.43[0.08,0.78] | -0.49[-0.72,-0.26] |
| **Andersen Model B Preoperative, outcome: 3 month recurrence, C in development 0.60*** | | | | | | | | | |
| All | 782/77 | 0.60[0.54-0.67] | 0.63[0.25,1.02] | -0.25[-0.49,-0.01] | 1733/164 | 0.67 | 0.59[0.51-0.66] | 0.50[0.01,1.00] | -0.20[-0.44,0.05] |
| AM | 0 | n/a | n/a | n/a | 159/11 | 0.64 | 0.58[0.39-0.78] | 0.55[-0.78,1.88] | -0.84[-1.47,-0.20] |
| RO | 0 | n/a | n/a | n/a | 611/59 | 0.65 | 0.61[0.41-0.81] | 0.72[-0.62,2.07] | 0.15[-0.55,0.85] |
| NE NL | 782/77 | 0.60[0.54-0.67] | 0.63[0.25,1.02] | -0.25[-0.49,-0.01] | 963/94 | 0.67 | 0.59[0.53-0.66] | 0.57[0.20,0.95] | -0.27[-0.49,-0.05] |

*corrected for optimism. AM= Amsterdam, NE NL= North East Netherlands, RO=Rotterdam, n/a= not available, C= concordance index, Mbc= Model based concordance.
